# First *De Novo* genome assembly and characterization of *Gaultheria prostrata*

**DOI:** 10.3389/fpls.2024.1456102

**Published:** 2024-10-29

**Authors:** Yan-Jun Lin, Xiao-Ya Ding, Yi-Wei Huang, Lu Lu

**Affiliations:** ^1^ School of Pharmaceutical Sciences, Yunnan Key Laboratory of Pharmacology for Natural Products, and Yunnan College of Modern Biomedical Industry, Kunming Medical University, Kunming, Yunnan, China; ^2^ Germplasm Bank of Wild Species, Kunming Institute of Botany, Chinese Academy of Sciences, Kunming, Yunnan, China; ^3^ University of Chinese Academy of Sciences, Beijing, China

**Keywords:** whole genome sequencing, gene annotation, evergreen shrubs, flow cytometry, microsatellite, SSR primers, transcription factors, phylogenetic relationships

## Abstract

*Gaultheria* Kalm ex L. (Ericaceae), a type of evergreen shrub, known as a natural source of methyl salicylate, possesses rich germplasm resources, strong habitat adaptability, significant ornamental value, and noteworthy pharmacological activities. However, due to the paucity of whole genomic information, genetically deep research in these areas remains limited. Consequently, we intend to obtain genome data through high-throughput sequencing, gene annotation, flow cytometry, transcription factors prediction and genetic marker analysis for a representative species of this genus, with *Gaultheria prostrata* selected for our study. In this study, we preliminarily obtained the genome of *G. prostrata* through next-generation sequencing methods. Utilizing 47.94 Gb of high-quality sequence data (108.95× coverage), assembled into 114,436 scaffolds, with an N50 length of 33,667 bp. The genome size assembled by SOAPdenovo, approximately 417 Mb, corresponded closely to predictions by flow cytometry (440 Mb) and *k*-mer analysis (447 Mb). The genome integrity was evaluated using BUSCO with 91%. The heterozygosity ratio was 0.159%, the GC content was 38.85%, and the repetitive regions encompassed over 34.6% of the genome. A total of 26,497 protein-coding genes have been predicted and annotated across Nr, Swissprot, GO, KEGG, and Pfam databases. Among these, 14,377 and 2,387 genes received functional annotation in Nr and Swissprot, respectively; 21,895, 24,424, and 22,330 genes were similarly annotated in GO, KEGG, and Pfam. Moreover, A total of 279,785 SSRs were identified and 345,270 primers for these SSRs were designed. Within the various nucleotide types of SSRs, AG/CT and AAG/CTT constituted the predominant dinucleotide and trinucleotide repeat types in *G. prostrata*. In addition, 1,395 transcription factors (TFs) from 75 TF families, 462 transcription regulators (TRs) from 33 TR families and 840 protein kinase (PKs) from 118 PK families were identified in this genome. We also performed phylogenetic analyses of *G. prostrata* and related species, including estimation of divergence times and expansion and contraction analyses, followed by positive selection analyses of orthologous gene pairs of *G. prostrata* and its close relative *Vaccinium corymbosum*. These results provide a reference for in-depth study of genus *Gaultheria*, contributing to future functional and comparative genomics analyses and providing supporting data for the development of molecular markers.

## Introduction

High-altitude woody plants, comprising both diploid and polyploid species, adapt through reinforced structures, deep roots, antifreeze proteins, elevated antioxidant enzyme activity, and systemic pathogen resistance, enabling them to withstand cold, UV exposure, and other environmental stresses (Nie et al., 2005; Couto and Zipfel, 2016; Ding et al., 2023a, 2023b). Like many montane woody plants, *Gaultheria* Kalm ex L. (Ericaceae), one of the most recently diverged taxa within Ericaceae with significant ploidy diversity, demonstrates remarkable adaptability. This genus is renowned not only for its rich germplasm resources, ornamental value, and notable pharmacological effects but also as a natural source of methyl salicylate, with strong adaptability to diverse habitats. The genus *Gaultheria* exhibits these traits largely due to its belonging to the Ericaceae family, renowned for its exceptional ecological adaptability, diverse symbiotic relationships, and resilience to environmental stressors. These qualities allow it to flourish and sustain biodiversity across a broad spectrum of extreme environments globally (Choudhary et al., 2021; BiologyInsights, 2024).

Notably, a lot of genome resources are still needed to reveal the complex evolutionary mechanisms of Ericaceae, but currently high-quality genome research is only focused on the two genera of *Rhododendron* (Yang et al., 2020) and *Vaccinium* (Cui et al., 2022). A large number of genome resources from other genera are urgently needed to supplement the research, especially the evolutionarily important and latest diverged *Gaultheria*, where there is no research on the whole genome. The analysis of *Gaultheria*’s evolutionary pattern mainly relies on chloroplast genomes or even nuclear gene fragments (Cheng et al., 2024). This limitation hinders the in-depth exploration of its genetic diversity, adaptive characteristics and evolutionary mechanisms. Hence, it is imperative to select representative species of the genus *Gaultheria* for high-throughput omics data to fill the genetic data gap of the genus, and deeply analyze the genome structure and gene expression pattern of the genus, so as to lay a solid foundation for exploring its diverse adaptation strategies. This dataset will then support broader research into the adaptive and evolutionary patterns of the Ericaceae family, further advancing knowledge in plant genomics.

A presumably diploid species within the Sympodial clade, *Gaultheria prostrata*, possesses the highest altitude distribution characterized by Rhododendron forests, Abies forest margins, shrublands, and rocky areas at altitudes between 4200 and 4800 meters in Himalaya-Hengduan Mountains, according to the sampling point records and reports from Flora of China (Wu et al., 2005). To be specific, *G. prostrata* is highly rich in methyl salicylate, accounting for about 70% of its own volatile oil content (Liu et al., 2013). This compound is not only widely used in the medical field (Li et al., 2016); more importantly, methyl salicylate’s functions as a signaling molecule, triggering systemic acquired resistance (SAR) (Chen et al., 2019b) and potentially enhancing defense abilities against environmental stresses typical of high altitudes, such as UV radiation and low temperatures, thereby likely aiding *G. prostrata* in adapting to high-altitude habitats. Deciphering *G. prostrata*’s genome will not only shed light the selection pressures on high-altitude diploid adaptive genes and their specific adaptive strategies, but also analyzes the role of its stress-resistant chemical composition in environmental adaptation.

To achieve the above goals, leveraging Next-Generation Sequencing (NGS) technology will be essential. *k*-mer analysis using NGS to enable access to a species’ genome size, GC content, repetition rate, and heterozygosity rates (Shan et al., 2020). In addition, this technology is also indispensable for analyzing the high-altitude adaptation mechanism of plants. For instance, a study utilized NGS technology to conduct a detailed analysis of the genomes of *Roscoea alpina* and *Roscoea purpurea*, exploring their adaptive differences at varying altitudes and revealing the genes and functions associated with high-altitude adaptation (Wang et al., 2024a). Another study employed NGS technology identifying key genes involved in *Saussurea obvallata* and *Rheum alexandrae*’s adaptation to high-altitude environments, such as DNA repair and antioxidant stress response genes (Zhang et al., 2023b). Besides, SSR (simple sequence repeat) marker is especially useful for researchers working on non-model organisms where genome resources might be limited, which helps in developing species-specific genetic markers. The combined use of NGS and SSR marker technologies will enable a comprehensive analysis of the unique high-altitude adaptation strategies of *G. prostrata*.

In summary, this study employed high-throughput sequencing technology to complete the sequencing, assembly, and annotation of the first genome of *G. prostrata* in the genus *Gaultheria*, providing foundational data for further exploration of its high-altitude adaptation. Subsequently, we utilized flow cytometry, genome survey analysis, and phylogenetic analysis to determine the genome size, and characteristics of this species, and to clarify their evolutionary relationships within the order Ericales. We also performed functional enrichment analysis on gene families involved in expansion/contraction and genes under positive selection. Then, we also designed several SSR markers to facilitate the construction of the genetic map of *Gaultheria* species. This provides important references for subsequent chromosome-level sequencing and supports in-depth research on functional genes and adaptations against stresses at high altitudes in *Gaultheria* species.

## Materials and methods

### DNA and RNA extraction from plant materials

#### DNA extraction

Three young leaves from an individual of *G. prostrata* were collected from tissue cultured plantlets at 25 ± 2°C, 16 h/8 h (light/dark) photoperiod, and 2000 lx illumination intensity. Leaf tissues were ground with liquid nitrogen and DNA was extracted using Biomarker Plant DNA Kit (Biomarker Technologies, Beijing, China). The red-boxed sample labeled “LL-2021-3,” representing *G. prostrata* (**Supplementary Figure S1**), shows a distinct band without smearing or blurring and no visible contaminants. The band intensity is appropriate, indicating that the DNA concentration is within an optimal range, which is typically indicative of high-quality DNA extraction (Sambrook and Russell, 2001). Additionally, we measured the DNA concentration using a Qubit 3.0 Fluorometer (Thermo Fisher Scientific), obtaining a result of 41 ng/µL. This indicates that the extracted DNA is of high quality and has an appropriate concentration, suitable for most molecular biology experiments (Thermo Fisher Scientific, 2014). The qualified genomic DNA is stored at -80°C for further use.

#### RNA extraction

Roots, stems, and leaves of *G. prostrata* were collected for RNA-Seq analysis, using three biological replicates for each tissue type. Immediately after harvested, samples were frozen in liquid nitrogen and stored at -80°C until extraction. Total RNA was extracted using the Trizol method (Honaas and Kahn, 2017).

### Genome size and ploidy level estimation by flow cytometry

#### Genome size estimation

Three young leaves from each plant were collected and immediately chopped in 0.8 mL of cold nuclei isolation buffer (45 mM MgCl_2_·6H_2_O, 20 mM MOPS, 30 mM Na-Citrate, 1% (w/v) PVP 40, 0.2% (v/v) Triton X-100, 10 mM Na_2_EDTA, 20 μL/mL *β*-mercaptoethanol, pH 7.5). The homogenate was filtered and transferred into the 1.5 mL tube using 40 μm nylon mesh. Then 50 mg/mL of DNA fluorochrome propidium iodide (PI) and 50 mg/mL of RNase were added and mixed gently with the samples. Before analysis, nuclei were stained on ice for 1 h away from light with occasional shaking. *Solanum lycopersicum* and *Zea mays* B73 was selected as internal references to detect the genomic DNA contents of *G. prostrata*. The fluorescence intensity of the emitted light of propidium iodide was detected using a BD FACScalibur flow cytometer (Becton Dickinson, NY, USA) on a sample of stained cell nucleus suspension using a 488 nm blue light excitation (Zhang et al., 2023a).

#### Ploidy level estimation

Flow cytometry is also a useful tool for estimating plant ploidy levels. This entails comparing the target species’ genome size with a known ploidy sample (karyotyped) or a consistent internal standard (Here, we selected two: *S. lycopersicum*, *Z. mays*), requiring karyotyping for at least one target species (Bourge et al., 2018; Pellicer et al., 2021). To deduce the ploidy level of only one species, *G. prostrata*, species with established ploidy levels and consistent internal standards were selected, including *G. griffithiana* (2n=4x=44) (Middleton and Wilcock, 1990), *G. crenulata* (2n=4x=44) (Middleton and Wilcock, 1990), *Vaccinium corymbosum* (2n=4x=44) (Yocca et al., 2023), and *V. macrocarpon* (2n=2x=44) (Yocca et al., 2023).

### Genome sequencing, transcriptome sequencing and genome evaluation

A genomic paired-end library with 270 bp insertions was performed on a BGISeq sequencing platform (MGI-2000) using the *G. prostrata* leaves. To minimize the impact of sequencing errors on the assembly, we used SOAPnuke (v1.6.5) software (Chen et al., 2018) to filter the raw sequencing data removing low quality reads with splice contamination and PCR duplication. Transcriptome sequencing is then conducted on both ends of the library using the DNBSEQ sequencing platform (BGI). The raw sequencing data undergo quality control using SOAPnuke (v1.6.5) too. During this step, reads containing adapter sequences, reads with more than 1% unknown bases (N), and low-quality reads (where more than 40% of bases have a quality score below 15) are filtered out. The result is a set of high-quality, clean data ready for downstream analysis. To estimate the genome size and heterozygosity and GC content of *G. prostrata*, Jellyfish (v2.1.4) software (Marcais and Kingsford, 2011) was used to analyze the *k*-mer depth distribution sequence of the filtered reads. Then the k-value of 21 was selected and GenomeScope2.0 software (Ranallo-Benavidez et al., 2020) was used to perform ploidy fitting to evaluate genomic characteristics. At the same time, utilizing *k*-mer (k = 21) histograms generated by KMC (v3.1.0) (Kokot et al., 2017), heterozygous *k*-mer pairs were analyzed with Smudgeplot (v0.2.5) (Ranallo-Benavidez et al., 2020) to estimate ploidy levels and infer genomic complexity. Lower (L) and upper (U) cut-off values were set based on *k*-mer coverage output from Genomescope 2.0, following recommendations in the Smudgeplot documentation (https://github.com/KamilSJaron/smudgeplot). *k*-mers falling below the lower cut-off or above the upper cut-off were discarded as errors. The lower cut-off value (L) was defined as (kcov/2) - 5, where kcov values were inferred from GenomeScope.

### Genome assembly, GC depth, SSR characteristics analysis and TFs/TRs/PKs identification

The clean reads obtained by filtering in the previous step were assembled by SOAPdenovo (v2.04) software (Luo et al., 2012). Subsequently, to evaluate the completeness of the genome, we used BUSCO v5.7.1 (Benchmarking Universal Single-Copy Orthologs) tools (Simao et al., 2015) with the embryophyta_odb10 dataset (creation date: 2024-01-08), which consists of 1614 Single-Copy Orthologs from 50 species. In order to measure the sequencing bias of *G. prostrata*, GC content and average sequencing depth were counted. The level of GC content is important for estimating plant genome size. The average GC sequencing depth was calculated from the assembled sequences along a 10-kb non-overlapping sliding window (Zhou et al., 2013). In addition, the microsatellite identification tool (MISA) (http://pgrc.ipk-gatersle-ben.de/misa/) was used to search for SSR loci on the assembled genome (Beier et al., 2017). The criteria for identifying SSR sequences are as follows: the minimum number of nucleotides repeats for mononucleotide repeat unit is 12 times, the number for dinucleotide repeat unit is 5 times, trinucleotide is 4 times, and the number for tetranucleotide, pentanucleotide, or hexanucleotide repeat unit is 3 times (Xu et al., 2022). Considering that mononucleotide repeat unit and compound repeat unit are not suitable as candidates for SSR marker development, these SSRs have been eliminated. Therefore, the main participants in primer design are di-, tri-, tetra-, penta-, hexa- nucleotides. Then, Primer 3.0 software was used to preliminarily design the primer pairs with parameters of final product length 80–250 bp, primer size of 18–25 bp, GC content of 35–70%, and annealing temperature of 50–60°C (Wang et al., 2018). After the design was completed, reduce_ssr.py (Yang et al., 2015) was used to remove the redundancy of the designed primer pairs, and 345,270 non-redundant primer pairs were obtained (The details of these primer pairs are provided in **Supplementary Table S1**). Finally, genome-wide transcription factors (TFs), transcriptional regulators (TRs), and protein kinases (PKs) were identified and classified using the iTAK (v2.02) software (Zheng et al., 2016).

### Gene prediction and annotation

We used RepeatModeler (v2.0.3) software (https://www.repeatmasker.org/RepeatModeler/) to construct a repetitive sequence library of *G. prostrata*, and used RepeatMasker (v4.1.4) software (https://www.repeatmasker.org/RepeatMasker/) to complete genomic repetitive sequence masking with the help of this repetitive sequence library. Then, we used ab initio prediction, homologous species prediction, and unigene gene prediction to predict and annotate the genes of *G. prostrata*. MAKER (v3.01.04) pipeline (Cantarel et al., 2008) were used for *de novo* prediction. It is a powerful analysis process that identifies repetitive sequences, compares ESTs and protein sequences to the genome, then performs *de novo* prediction using SNAP (v2006-07-28) software (Korf, 2004), Augustus (v3.3.3) software (Stanke et al., 2006), and GeneMark-ES (v4.69) software (Lomsadze et al., 2005), finally integrates the results from all three software to ensure the reliability of the results. In addition, MAKER can be trained continuously, and the initial output can be used as an input file in the algorithm for re-training the gene prediction model, thus obtaining a higher quality gene model. GeMoMa (v1.9) software (Keilwagen et al., 2018) was used to predict homologous data, and TransDecoder (v5.5.0) software (https://github.com/TransDecoder/TransDecoder/wiki) was used to predict transcription data. After completing all the above predictions and annotations, the final results were integrated with EVidenceModeler (v1.1.1) pipeline (https://github.com/EVidenceModeler/EVidenceModeler/wiki) (Altschul et al., 1990). Ultimately, we used GFAP (v3.1) (Xu et al., 2023), a program for functional annotation of plant genes, to perform GO, KEGG, Pfam, nr, and swissprot functional annotations on *G. prostrata*, respectively.

### Phylogenomic analysis, divergence time estimation and expansion and contraction analysis

We used OrthoFinder (v.2.5.5) (Emms and Kelly, 2015) with default parameters to obtain common single-copy genes from the genomes of *G. prostrata*, six species from the order Ericales (*Actinidia chinensis*, *Rhododendron williamsianum*, *Vaccinium corymbosum*, *Aegiceras corniculatum*, *Diospyros kaki*, *Camellia sinensis*), one species from the family Brassicaceae (*Arabidopsis thaliana*), and one species from the family Poaceae (*Oryza sativa*). These single-copy genes were then used to reconstruct the phylogenetic tree, with *O. sativa* selected as the outgroup. Multiple amino acid sequence alignment was performed using Muscle (v5.1) (Edgar, 2004), followed by extraction of conserved sequences from the aligned files using Gblocks (0.91b) (Talavera and Castresana, 2007) and merging them with seqkit. ProtTest (v3.4.2) (Darriba et al., 2011) was used to predict the appropriate amino acid substitution model, identifying the JTT+I+G+F model as the best fit according to both AIC and BIC criteria. This model was then used to build the tree using RAxML (v8.2.12) (Stamatakis, 2014) with 1000 bootstrap replicates.

Divergence times were estimated using the MCMC Tree method in the PAML package (v.4.10.7) with five calibration constraints [refer to TimeTree (Kumar et al., 2017): http://www.timetree.org and Fossil calibration database (Ksepka et al., 2015): https://fossilcalibrations.org]. These included a fossil constraint for the most recent common ancestor (MRCA) of *O. sativa* and *A. thaliana* (142.1 to 163.5 million years ago, Mya); a fossil constraint for the MRCA of *A. thaliana* and *A. corniculatum* (111.4 to 123.9 Mya); a fossil constraint for the MRCA of *C. sinensis* and *A. chinensis* (82.8 to 106 Mya); a fossil constraint for the MRCA of R*. williamsianum* and Vaccinioideae (45.5 to 76.9 Mya); and a fossil constraint for the MRCA of *V. corymbosum* and *G. prostrata* (4.6 to 59.2 Mya).

CAFE software (v5.1.0) was used to combine the results of the previous phylogenetic analysis to screen out gene families that showed expansion or contraction (Mendes et al., 2020) with parameters set to 10 threads and a significance threshold of P = 0.05. At the same time, Chiplot (Xie et al., 2023) was used to plot pie charts of the proportion of expansion and contraction genes. Those significantly expanded and contracted gene families in *G. prostrata* were then subjected to functional enrichment analysis using Gene Ontology (GO) and Kyoto Encyclopedia of Genes and Genomes (KEGG) databases to elucidate their potential roles and biological functions.

The species photos were drawn by DALL-E 2 (https://openai.com/index/dall-e-2/)

### Ka/Ks positive selection analysis

Initially, we employed WGD software (v1.1) to identify orthologous gene pairs between *G. prostrata* and its closely related species, *V. corymbosum*, for whose genomes were available. Subsequently, ParaAT software (v2.0) was used to align homologous sequences and convert amino acid sequences into CDS sequences (coding DNA sequences). Next, KaKs_Calculator software (v2.0) was employed to calculate Ka and Ks values. Finally, a custom Python script was used to screen genes with ω > 1 and perform GO and KEGG databases functional annotations.

## Results

### Genome size and ploidy level estimation by flow cytometry

#### Genome size estimation

**Figure 1:**
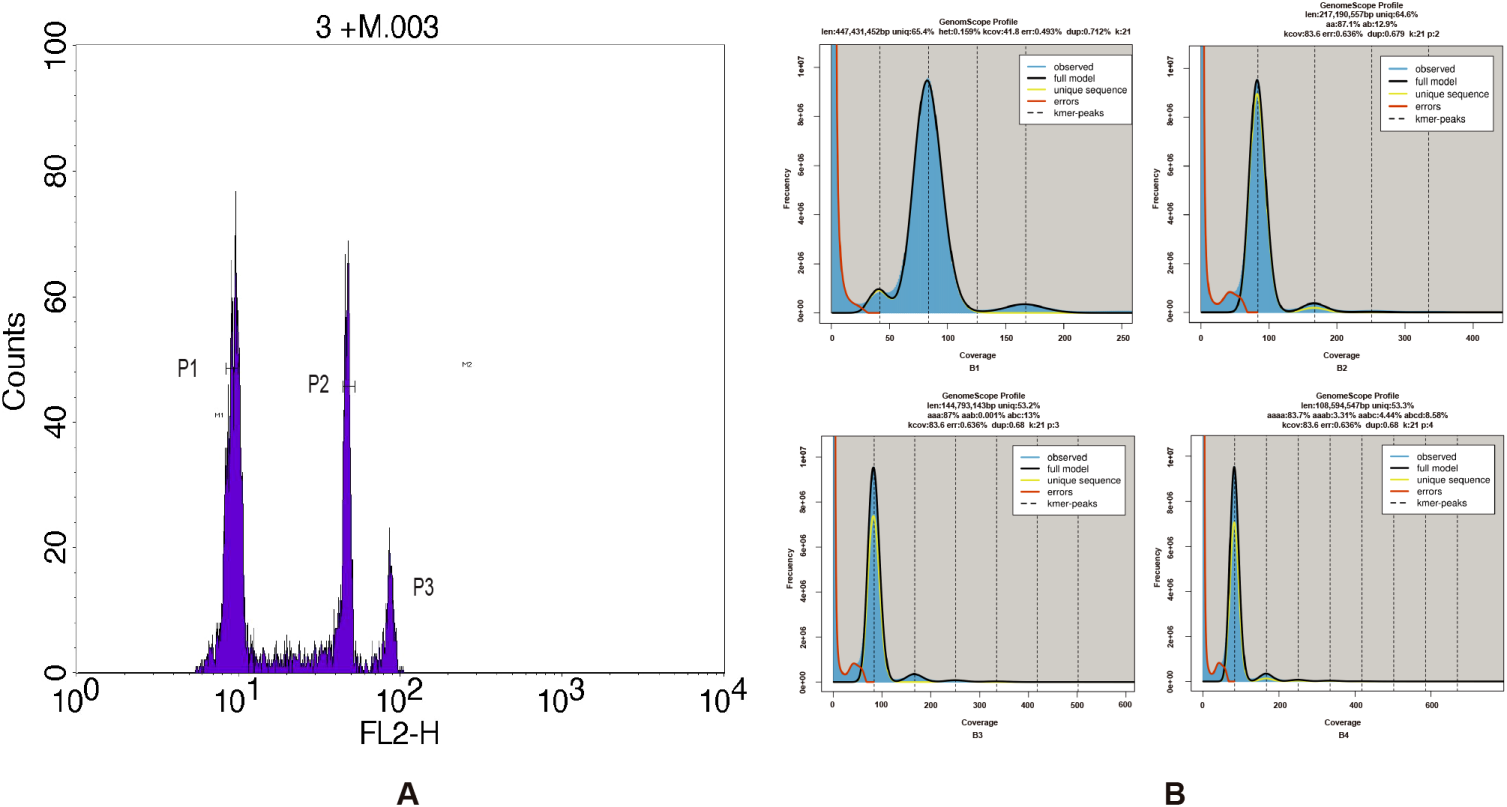
Flow cytometry and *k*-mer analysis of the *Gaultheria prostrata* genome. **(A)** Histogram of relative fluorescence intensity derived from nuclei isolated from *Z. mays*, *S. lycopersicum* and *G. prostrata* processed simultaneously. The horizontal axis represents the fluorescence intensity, and the vertical axis represents the number of cells corresponding to each fluorescence intensity. Peak1, Peak2, and Peak3 correspond to the fluorescence intensity of *G*. *prostrata*, *S. lycopersicum*, and *Z. mays* samples, respectively. **(B)** *k*-mer (k = 21) distribution calculated by Genomescope (B1: GenomeScope 1.0; B2–B4: ploidy fitting by GenomeScope 2.0). The observed *k* mer distribution is shown as blue bars. The black line models the distribution, excluding *k*-mer errors (red line) and extends to the maximum *k*-mer coverage specified in the model (yellow line). Abbreviations are as follows: ‘len’ for estimated genome length, ‘uniq’ for the unique portion of the genome (nonrepetitive elements), ‘het’ for genome heterozygosity, and ‘err’ for the sequencing error rate.

The flow cytometric analysis produces a high-resolution histogram with mean values of *G. prostrata* with the internal references including *Z. mays* and *S. lycopersicum*. From the results, it can be seen that the internal fluorescence intensities of *Z. mays* and *S. lycopersicum* are 45.19 and 21.62, respectively. The CVs of *Z. mays*, *S. lycopersicum* and *G. prostrata* were 6.09%, 8.53% and 11.80%. Compared to them, the fluorescence intensity of *G. prostrata* is 8.99 and 10.77, respectively (**Figure 1A**). Besides, the DNA content of *G. prostrata* is 0.46 pg (1C value) or 0.92 pg (2C value), with an estimated genome size of approximately 440 Mb.

#### Ploidy level estimation

Tetraploid *G. griffithiana* and *G. crenulata* have DNA contents of 2.02 pg (~970 Mb) and 2.07 pg (~990 Mb), respectively. Tetraploid *Vaccinium corymbosum* and diploid *V. macrocarpon*’s genome sizes are ~980 Mb and ~500 Mb, respectively. The DNA content and genome size of tetraploid *G. griffithiana* and *G. crenulata* are about double those of *G. prostrata*, within the same genus. In comparison, tetraploid *V. corymbosum*’s genome is roughly twice, and diploid *V. macrocarpon*’s genome size closely matches *G. prostrata*’s. Therefore, *G. prostrata* is inferred to be a diploid species.

### Genome sequencing and genome evaluation

**Figure 2:**
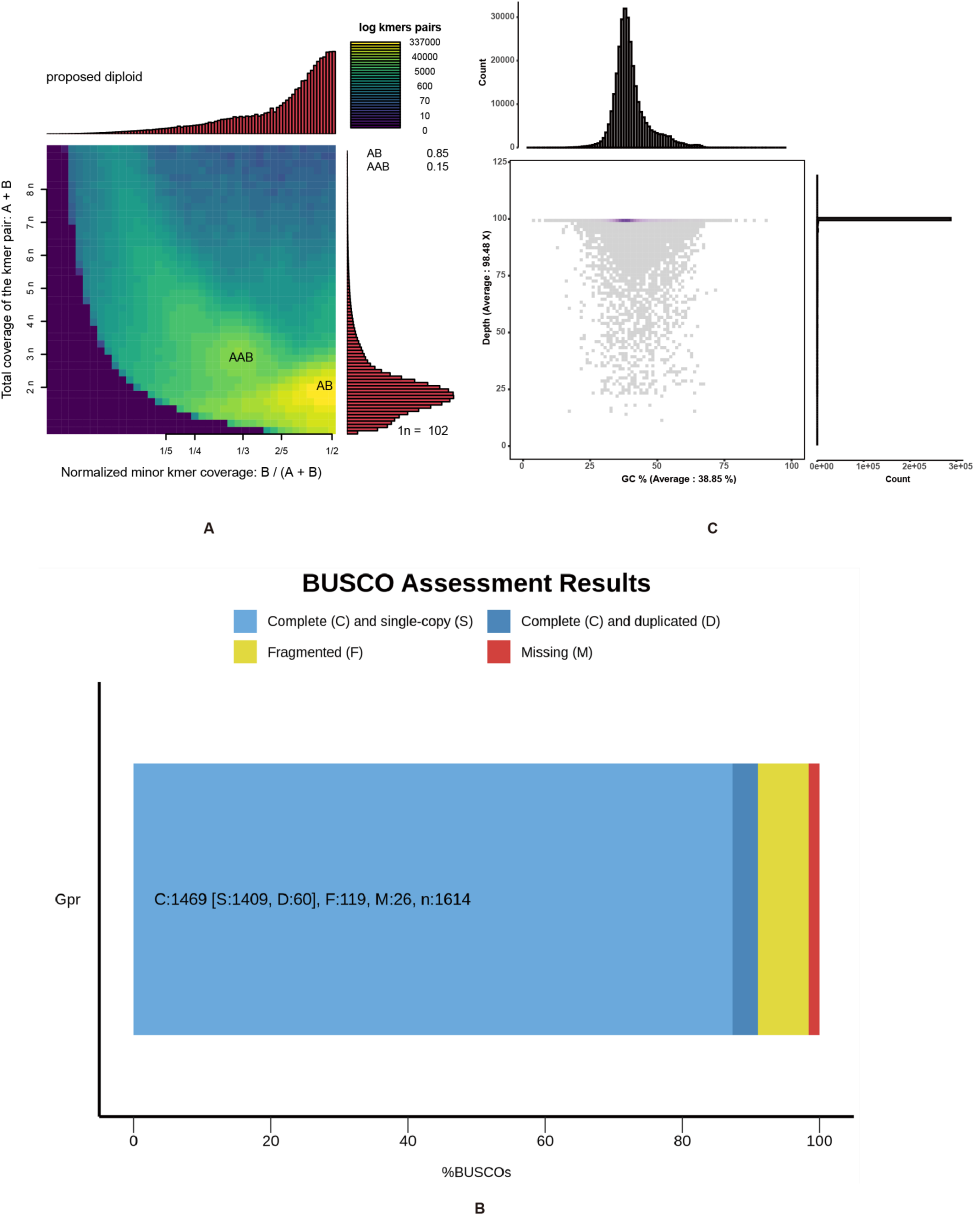
Smudgeplot analysis, BUSCO evaluation and GC-depth distribution of the *Gaultheria prostrata* genome. **(A)** Two-dimensional heat maps were constructed using Smudgeplot (k = 21) to illustrate the ploidy predictions from clean reads. Color intensity indicates the approximate number of *k*-mers per bin, ranging from purple (low) to yellow (high). Estimated ploidies are displayed in the upper left corner of each graph, along with the likelihood of various ploidy levels. The top histogram shows the frequency distribution of different minor *k*-mer coverage ratios (B/(A + B)). The right histogram shows the frequency distribution of different total coverages (A + B). **(B)** The BUSCO assessment of genome assembly completeness was conducted. The BUSCO assessment was executed in genome mode using Miniprot as the gene predictor. The x-axis represents the percentage of various types of BUSCOs, and the y-axis indicates the genome names. **(C)** The figure comprises two histograms and one scatter plot. The upper histogram shares the x-axis with the scatter plot, and the histogram on the right shares the y-axis with the scatter plot. The x-axis of the scatter plot represents GC content, and the y-axis represents sequencing depth; The histogram above the scatter plot displays the count of reads by varying GC contents in the *G*. *prostrata* genome; The histogram to the right of the scatter plot illustrates the counts of reads by different sequencing depths in the *G*. *prostrata* genome.

The genome of *G. prostrata* was sequenced by BGISeq sequencing platform (MGI-2000). After filtering with SOAPnuke, 47.94 Gb high-quality data were obtained. The Q20 was 94.27% and the Q30 was 82.64%. Besides, sequencing depth was 108.95×. All the clean data were used for *k*-mer analysis (the total *k*-mer number was 4,257,951,935), By simulating using Jellyfish software, we performed *k*-mer frequency distribution analysis with K = 21. By calculating using the formula that Genome size=*k*-mer num/peak depth, the genome size of it was estimated at around 447Mb. **Figure 1B** (B1) shows the results of GenomeScope software analysis: there are three obvious peaks and the horizontal coordinates corresponding to the peaks are in a multiplicative relationship and there is a certain degree of heterozygosity and the proportion of repetitive sequences, of which the one at depth=42 is a heterozygous peak, the one at depth=83 is the main peak, and the repetitive peak is at depth=166. The heterozygous peak is shown to be at approximately half the depth of the major peak, indicating possible diploidy. A similar pattern is observed in **Figure 1B** (B2), supporting the inference of diploidy. Although both **Figure 1B** (B3) and (B4) showed one main peak and multiple other peaks, they had no clear polyploidy characteristics, which made the final ploidy inference biased towards diploidy. Besides, the proportion of repeats in the *G. prostrata* genome was 34.6% and the heterozygosity was 0.159% (**Figure 1B**), and it suggested that the genome was not a highly complex one. The ratio of heterozygous *k*-mer pairs in Smudgeplot also indicates that *G. prostrata* may be a diploid, with an average of 85% of *k*-mer pairs belonging to the AB type. Although there is still AAB type, it accounts for a relatively small proportion of only 15% (**Figure 2A**).

### Genome assembly, GC depth, SSR characteristics analysis and TFs/TRs/PKs identification

SOAPdenovo software was used for *de novo* assembly with a 21-mer selection. A total of 417,467,430 scaffolds were assembled into a final genome sequence of 410 Mb with a scaffold N50 length of 33,667 bp (**Table 1**). The BUSCO genome completeness assessment indicates that 91.0% of the assembly is complete (C: 1,469), with 1,409 (87.3%) being complete and single-copy BUSCOs (S), 60 (3.7%) being complete and duplicated BUSCOs (D), and only 119 (7.4%) being fragmented BUSCOs (F). Out of a total of 1,614 BUSCOs searched, only 26 (2.5%) were missing (M) (**Figure 2B**). The number of complete (C) BUSCOs is significantly higher than the number of fragmented (F) and missing (M) BUSCOs, suggesting a high-quality genome assembly (More evaluation details are shown in **Supplementary Table S2**). Besides, the sequences produced by *de novo* assembly were similar with the genome size estimated by *k*-mer analysis (447 Mb) and flow cytometry (440 Mb), again indicating that the genome of *G. prostrata* might be a diploid with a relatively simple structure. Additionally, the GC content and average depth of *G. prostrata* genome were then calculated with 10-kb non-overlapping sliding window (**Figure 2C**). The GC content of it was 38.85%, with a mid-GC content between 30 and 50%. Meanwhile, the depth of sequencing was 98.48×, which indicated the high quality of our sequence.

**Table 1:** Summary for the *de novo* assembly of the *Gaultheria prostrata* genome.

Statistical item	Contig	Scaffold
Total length (bp)	417,380,110	417,467,430
Number of sequences	115,589	114,436
N50 length (bp)	31,895	33,667
N60 length (bp)	25,031	26,309
N70 length (bp)	18,325	19,408
N80 length (bp)	11,676	12,386
N90 length (bp)	4,170	4,446
N100 length (bp)	128	128
GC content (%)	38.85	
Depth of sequencing (×)	98.48	

**Figure 3:**
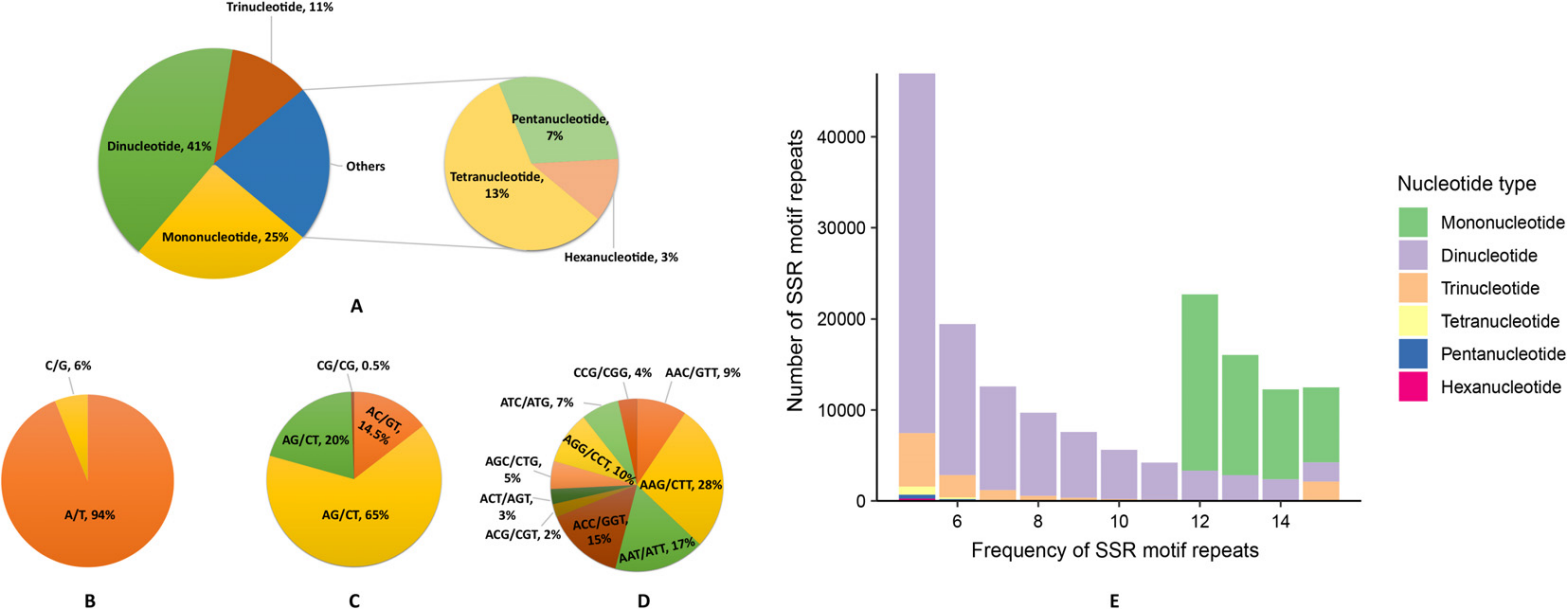
Characteristics and frequency of SSR motif repeats in the *Gaultheria prostrata* genome. **(A)** Percentage of different nucleotide types. **(B)** Percentage of different motifs in mononucleotide repeats. **(C)** Percentage of different motifs in dinucleotide repeats. **(D)** Percentage of different motifs in trinucleotide repeats. **(E)** Number of various SSR motif repeats in different repeat frequencies. (**A, E**: Various colors represent different nucleotide types; **B–D**: Colors differentiate nucleotide types.).

A total of 279,785 SSRs were identified using the Perl script MISA, Dinucleotide repeats (41.33%) constitute the most abundant type, followed by mono- (25.22%), tetra- (12.76%), tri- (11.37%), penta- (6.69%), and hexa- (2.62%) nucleotide repeats. Among them, 463 repeat units were identified, with AG/CT (64.82%) as the predominant type among the dinucleotide repeat motifs, followed by AT/AT (20.11%), AC/GT (14.51%), and CG/CG (0.56%). The three most abundant trinucleotide motifs were AAG/CTT (27.66%), AAT/ATT (17.01%), and ACC/GGT (14.91%) (**Figures 3A–D**); the four most abundant tetranucleotide motifs were AAAT/ATTT (28.74%), AAAG/CTTT (15.71%), ACCC/GGGT (9.93%), and AATT/AATT (5.50%). Besides, the most abundant pentanucleotide repeats are AAAAT/ATTTT (16.09%), AAAAG/CTTTT (14.50%) and AAACC/GGTTT (8.74%). The most abundant hexanucleotide repeats are AAAAAT/ATTTTT (15.31%) AAAAAG/CTTTTT (11.45%) and AAAAAC/GTTTTT (6.17%)(**Supplementary Table S3**). Moreover, the frequency of repeats for different types of SSR loci predominantly ranges between 5 and 15 (Shi et al., 2016b). We plotted the frequency of SSR motif repeats (**Figure 3E**), revealing that the highest percentage is of dinucleotides, followed bymononucleotides, and then trinucleotides. Mononucleotides predominantly occur in the 12-15 frequency range, while dinucleotides are mostly found in the 5-10 frequency range and trinucleotides are in the 5-10 and 15 frequency range. (**Supplementary Table S4**)

**Figure 4:**
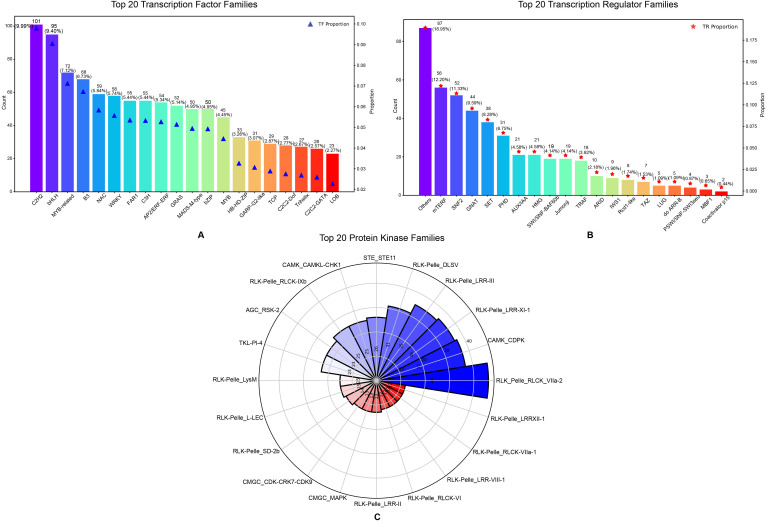
Top twenty TF families, TR families and PK of *Gaultheria prostrata* genome. **(A)** The bar chart illustrates the top 20 transcription factor families, classified by their counts and proportions. The x axis enumerates the various transcription factor families, while the left y-axis represents the count, and the right y-axis denotes the proportion. **(B)** The bar chart displays the top 20 transcription regulator families based on their counts and proportions. The x-axis lists the different transcription regulator families, while the y-axis on the left shows the count, and the y-axis on the right indicates the proportion. **(C)** The Nightingale Rose Plot (also known as a Radar Chart) illustrates the top 20 protein kinase families. Each sector represents a different protein kinase family, with the radius of the sector proportional to the number of members in that family. The intersection of the radial lines and the circle denotes the tick marks for the gene family count, with the number of each family also marked in the corresponding sector. **(A–C)** use color coding to distinguish between families.

In addition, 1,395 transcription factors (TFs) from 75 TF families, 462 transcription regulators (TRs) from 33 TR families and 840 protein kinase (PKs) from 118 PK families were identified in this genome (**Supplementary Tables S5**, **S6**; **Figures 4A–C**):

**Figure 4A** shows the top 20 transcription factor families by quantity, with C2H2 being the most abundant (101 members, 9.99%), followed by bHLH (95 members, 9.40%) and MYB-related (72 members, 7.12%). Families like C2H2, B3, NAC, and WRKY are involved in environmental adaptation, while C2H2, bHLH, MYB, and others regulate growth and development (Puranik et al., 2012). Their functions span stress response, developmental regulation, and signal transduction. **Figure 4B** shows the distribution of the top 20 transcriptional regulator families, with “Others” comprising 87 genes (18.95%) and mTERF, SNF2, GNAT, and SET families following, ranging from 12.20% to 8.28% of the total. These regulators play key roles in gene expression and chromatin structure, where mTERF, SNF2, and HMG primarily control mitochondrial and nuclear gene regulation, while GNAT, SET, PHD, and Jumonji influence gene expression through histone modifications (Kouzarides, 2007). AUX/IAA proteins act as repressors in the auxin signaling pathway, regulating plant growth (Mockaitis and Estelle, 2008). **Figure 4C** shows the distribution of the top 20 protein kinase families, with RLK-Pelle_RLCK-VIIa-2 being the most abundant (46 members), followed by CAMK_CDPK (37 members) and RLK-Pelle_LRR-XI-1 (36 members). These families play key roles in plant signal transduction pathways, regulating growth, defense, and stress responses. Specifically, RLK-Pelle is involved in development and defense, CDPKs mediate stress through calcium signaling, and STE11 participates in the MAPK pathway for cell division and stress response (Gish and Clark, 2011; Schulz et al., 2013).

### Gene prediction and annotation

After constructing the repetitive sequence library of *G. prostrata* by RepeatModeler, the *G. prostrata*-families.fa was identified by using ReapeatMasker, and the results showed that the repetitive sequences accounted for 45.04%, with a total length of 188,030,998 bp, of which the interspersed repeats accounted for 43.5%. Wherein, the proportion of LINE (long interspersed nuclear elements) (1.72%) and SINE (short interspersed repeated sequence) (0.75%) were lower than LTR (long terminal repeats) (11.71%) and DNA transposons (4.78%). Besides, in tandem repeat sequences, satellites accounted for 0.14%; low complexity (0.16%) and simple repeats accounted for 0.88% (**Table 2**). Secondly, an ab initio prediction wasmade using MAKER. A total of 599,422 genes were annotated by Augustus, 809,961 genes by SNAP, and 1,435,945 genes by Genemark. Then, homologous gene annotation was completed using GeMoMa and a total of 73,995 genes were annotated. Meanwhile, 25,020 transcriptome genes were predicted using TransDecoder. Finally, all predicted genes were integrated using EVidenceModeler and 34,909 annotated genes were obtained. Subsequently, functional gene annotation was performed using the results from EVidenceModeler integration.

**Table 2:** Major types of repeat elements identified in the *Gaultheria prostrata* genome.

Repeat class	Number of elements	Length occupied	Percentage of sequence
Retroelements	185,611	59,180,031 bp	14.18%
SINEs	23,033	3,110,672 bp	0.75%
Penelope	0	0	0
LINEs	20,718	7,180,507 bp	1.72%
CRE/SLACS	0	0	0
L2/CR1/Rex	808	221,811 bp	0.05%
R1/LOA/Jockey	348	80,698 bp	0.02%
R2/R4/NeSL	576	96,869 bp	0.02%
RTE/Bov-B	4,300	1,175,974 bp	0.28%
L1/CIN4	13,976	5,404,488 bp	1.29%
LTR elements	141,860	48,888,852 bp	11.71%
BEL/Pao	188	50,726 bp	0.01%
Ty1/Copia	48,622	16,348,615 bp	3.92%
Gypsy/DIRS1	59,901	25,667,125 bp	6.15%
Retroviral	1,963	323,908 bp	0.08%
DNA transposons	96,356	19,955,967 bp	4.78%
Hobo-Activator	53,634	10,213,902 bp	2.45%
Tc1-IS630-Pogo	8,166	1,648,465 bp	0.39%
En-Spm	0	0	0
MULE-MuDR	11,886	3,333,689 bp	0.80%
PiggyBac	0	0	0
Tourist/Harbinger	6,935	1,538,130 bp	0.37%
Other(Mirage, P-element, Transib)	0	0	0
Rolling-circles	2,293	821,731 bp	0.20%
Unclassified	634,462	102,475,106 bp	24.55%
Total interspersed repeats		181,611,104 bp	43.50%
Small RNA	25,350	3,369,409 bp	0.81%
Satellites	2,549	565,329 bp	0.14%
Simple repeats	98,396	3,668,654 bp	0.88%
Low complexity	14,724	681,631 bp	0.16%

A total of 26,497 protein-coding genes were predicted and annotated in Nr, Swissprot, GO, KEGG and Pfam databases using GFAP. Among these genes, 14,377 and 2,387 genes were functionally annotated on Nr and Swissprot, respectively; 21,895, 24,424 and 22,330 genes were functionally annotated on GO, KEGG and Pfam separately. (The integration annotation files for the five databases and the separate GO, KEGG, and Pfam databases are detailed in **Supplementary Tables S7**–**S12**).

**Figure 5:**
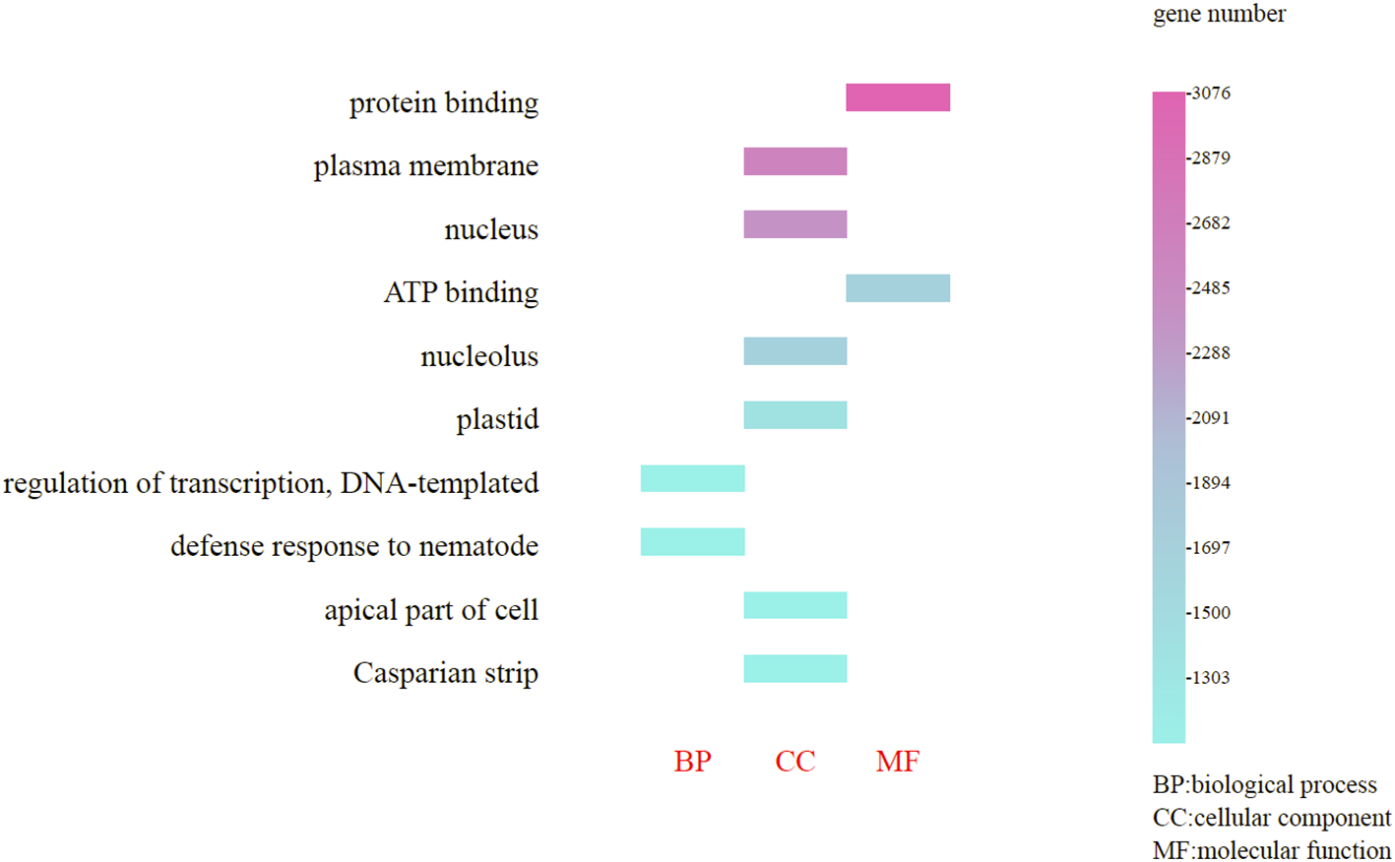
GO functional classification of the *Gaultheria prostrata* genome. The horizontal axis represents the three fundamental GO categories: biological Process (BP), cellular component (CC) and molecular function (MF); Captions on the left side of the chart correspond to specific terms for BP, CC, or MF; The vertical axis, located on the right, indicates the number of genes enriched for each term.

The genes annotated in the GO database are categorized into three primary groups: cellular component processes (58.20%), molecular functions (25.40%), and biological processes (16.40%); these percentages indicate the proportion of all enriched genes within each category. Furthermore, within the cellular component processes, the most enriched genes were those associated with the plasma membrane (2,879 genes) and nucleus (2,682 genes); in the molecular function category, protein binding (3,076 genes) and ATP binding (2,485 genes) were predominant; and in the biological processes category, regulation of transcription, DNA-templated (1,894 genes) and defense response to nematodes (1,697 genes) were notably enriched (**Figure 5**). (Refer to **Supplementary Tables S8**, **S9** for detailed functional information about specific genes.)

**Figure 6:**
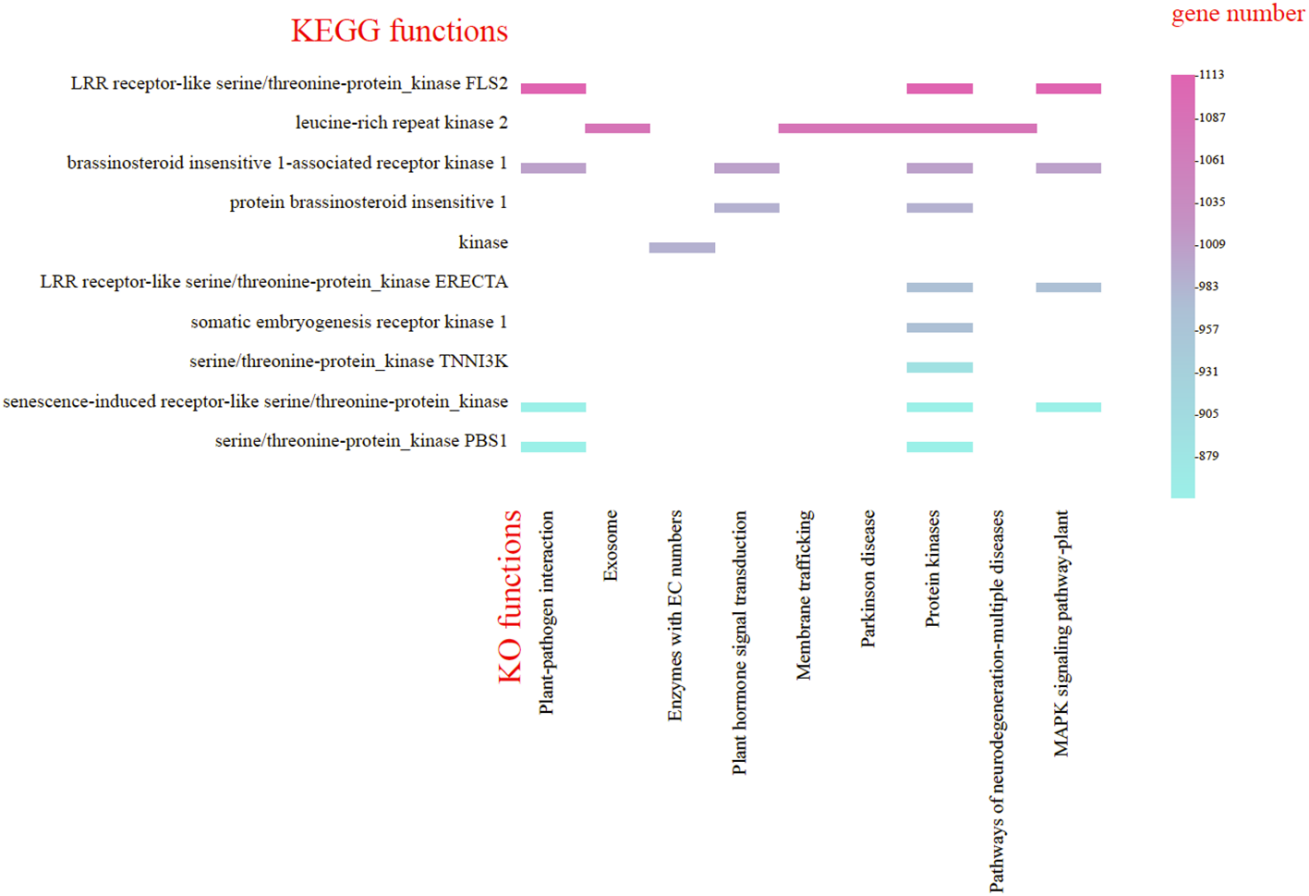
KEGG functional classification of the *Gaultheria prostrata* genome. The horizontal axis represents the highly enriched KO pathway terms; Captions on the left side of the chart to kinases involved in the KO pathway terms. The vertical axis, located on the right side of the chart, indicates the number of genes enriched for each term.

Moreover, the genes annotated in the KEGG database were divided into nine categories, predominantly featuring protein kinase genes associated with plant-pathogen interaction, plant hormone signal transduction and MAPK signaling pathway-plant. The largest group (16.6%) was associated with the MAPK signaling pathway, featuring protein kinases like FLS2, ERECTA, and BRI1. Plant-pathogen interaction (16.2%) included kinases such as PBS1 and BRI1, while hormone signal transduction (8.58%) involved BRI1-related kinases (**Figure 6**). (Refer to **Supplementary Tables S10**, **S11** for detailed functional information about specific genes.)

### Phylogenomic analysis, divergence time estimation and expansion and contraction analysis

We conducted phylogenomic and molecular dating analyses based on 12 single-copy genes derived from the genomes of *G. prostrata*, six species from the order Ericales (*A. chinensis*, *R. williamsianum*, *V. corymbosum*, *A. corniculatum*, *D. kaki*, *C. sinensis*), one species from the family Brassicaceae (*A. thaliana*), and one species from the family Poaceae (*O. sativa*). *G. prostrata* was assigned to the subfamily Vaccinioideae within the family Ericaceae, with a strongly supported topology [((Vcor,Gpro)100,Rwil)] (**Supplementary Figure S2**).

**Figure 7:**
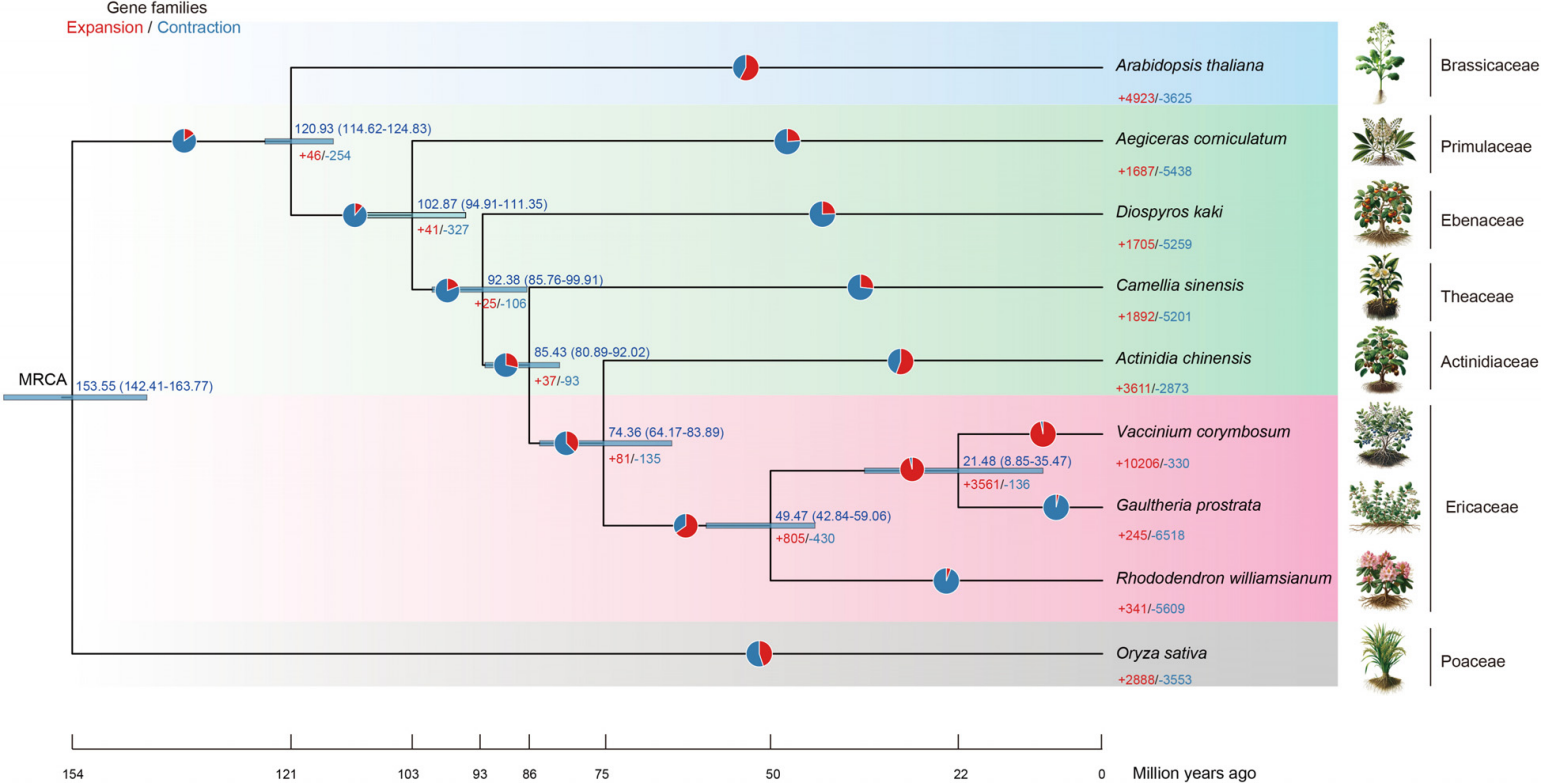
The time-calibrated phylogenetic tree for Ericales. The chronogram illustrates the divergence times within the order Ericales, encompassing families such as Primulaceae, Ebenaceae, Theaceae, Actinidiaceae, and Ericaceae. The species photos were drawn by DALL-E 2 (https://openai.com/index/dall-e-2/). The pie chart represents the proportion of expansion/contraction genes.

Based on our selected species, the crown age of the order Ericales is estimated to be approximately 102.87 Ma (with a 95% highest posterior density (HPD) interval of 94.91–111.35 Ma) (**Figure 7**). This estimate is supported by molecular clock calibration studies from Bell et al. (2010); Herting et al. (2023), and Magallón and Castillo (2009), and aligns with the diversification events of other angiosperms (Magallón and Castillo, 2009; Bell et al., 2010; Herting et al., 2023). The families Primulaceae and Ebenaceae originated in the Late Cretaceous (Late Mesozoic), with the divergence time of Ebenaceae estimated at ∼92.38 Ma (95% HPD interval: 85.76–99.91 Ma). According to our species sampling, the stem age of the family Ericaceae dates back to ∼49.47 Ma (95% HPD interval: 42.84–59.06 Ma), while the divergence time between *V. corymbosum* and *G. prostrata* in the subfamily Vaccinioideae is approximately 21.48 Ma (95% HPD interval: 8.85–35.47 Ma) (**Figure 7**; **Supplementary Figure S3**).

This phylogenetic tree illustrates gene families of expansion and contraction within the Ericales, focusing on *G. prostrata*. It shows a substantial contraction gene (-6,518 genes) with minimal expansion (+245 genes), suggesting significant gene loss or streamlining, possibly due to specific environmental adaptations. In contrast, *V. corymbosum* exhibits a large gene expansion (+10,266), indicating different evolutionary pressures. The broader Ericales lineage, including *R. williamsianum*, also shows notable gene contraction (-5,609), reflecting diverse evolutionary strategies across the order (**Figure 7**).

The pathways related gene expansion in *G. prostrata*, such as Protein phosphatases and associated proteins and MAPK signaling pathway (**Supplementary Table S13**), indeed play significant roles in cell signaling, pathogen defense, and substance transport, which are essential for adapting to extreme environments like high altitudes. Expansion genes support processes such as xenobiotic transmembrane transport, responses to organonitrogen compound and hormone and auxin transport (**Supplementary Table S14**), which contributing to adaptation to harsh conditions like low oxygen and high UV radiation. Conversely, significant gene contraction reflects an adaptive strategy to conserve resources by reducing reliance on non-essential metabolic pathways, such as Cytochrome P450, secondary metabolite synthesis, and cellular responses to light intensity (**Supplementary Tables S15**, **S16**). This reduction streamlines metabolic processes, improving efficiency and adaptation to the resource-limited and harsh conditions typical of high-altitude environments.

### Ka/Ks positive selection analysis

**Figure 8:**
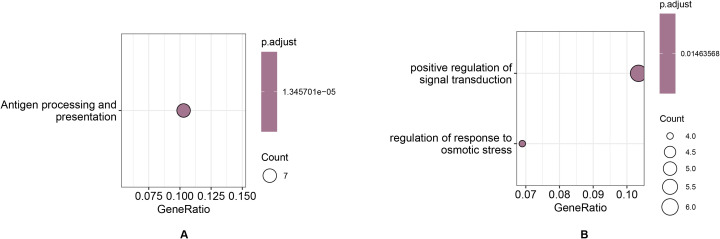
Functional enrichment of positive selection genes in *Gaultheria prostrata* related to **(A)** Antigen processing and presentation and **(B)** Positive regulation of signal transduction and Response to osmotic stress, highlighting enhanced immune and stress response mechanisms adapted to high-altitude environments.

The positively selected orthologous gene pairs of *G. prostrata* and *V. corymbosum* identified through positive selection analysis have similar functions, so only the positively selected genes for G. prostrata are discussed here as representatives. As shown in **Figure 8A** and **Supplementary Table S17**, the significantly enriched positive selection genes related to Antigen processing and presentation are prevalent or active in *G. prostrata*. This could suggest an adaptive immune or stress response mechanism tailored to high-altitude environments where these plants encounter unique pathogens or extreme stresses. In **Figure 8B** and **Supplementary Table S18**, pathways related to positive regulation of signaling, including osmotic stress response and signal transduction, are enriched. These enriched pathways reflect strong signaling capabilities in both species, underscoring their ability to maintain cellular functions and growth while adapting to the harsh, variable conditions of high-altitude environments. This highlights their robust stress response and signaling mechanisms, crucial for survival under extreme environmental pressures.

## Discussion

Woody plants are crucial for ecological protection in high-altitude habitats. However, genomic research on these plants is relatively scarce (Saikia et al., 2017). In this study, a genome sequencing draft was assembled using next-generation high-throughput sequencing for the woody plant *G. prostrata* genome, aiming to obtain accurate short read data, which will serve as an important reference for obtaining further third-generation data. Owing to its high accuracy and low error rate, this data has advantages in error correction and calibration of third-generation sequencing data. In-depth genomic research on *G. prostrata* aims to discover genes with specific functions and adaptability laying the foundation for analyzing the genetic evolutionary patterns of *Gaultheria* species and providing support for the conservation of genetic diversity, ecological adaptation, and functional trait evolution research of this species.

Flow cytometry is a standard method for rapid C-value acquisition and genome size prediction (Zhu et al., 2012). *k*-mer analysis of genome sequencing data is also used to assess characteristics such as size, heterozygosity, repetitive sequences and GC content (Zhou et al., 2023). Given the rapid advancement of NGS technology, this study integrates flow cytometry and *k*-mer analysis to enhance genome size prediction reliability. The genome size of *G. prostrata*, as determined by *k*-mer deep analysis (447Mb), aligns closely with the results from flow cytometry (440 Mb). Furthermore, the draft genome size for the initial assembly (410 Mb) correlates well with these predictions, thereby validating their accuracy. Numerous studies have concurrently assessed genome size using above three methods, highlighting their widespread use and significance (Pflug et al., 2020; Pfenninger et al., 2022). Besides, flow cytometry analysis reveals that the genome size of *G. prostrata* is nearly half of the tetraploid species *G. crenulata* and *G. griffithiana*, indicating that *G. prostrata* is likely a diploid species. On this basis, we conducted Smudgeplot analysis, which also further supported that *G. prostrata* is an AB diploid rather than a polyploid. Furtherly, cytological studies should be conducted in the future to confirm the chromosome number and verify the ploidy type. In general, the genus *Gaultheria* has both polyploid and diploid (Middleton’s literature suggests that the base chromosome number of the *Gaultheria* Kalm ex L. (Ericaceae) taxon is x=11). In the future, an in-depth exploration of the causes of polyploidy formation or diploidy maintenance in different species can be conducted based on this diploid framework.

Additionally, genome assembly quality is significantly influenced by factors such as heterozygosity and repetition rate (Bi et al., 2019). Assembly becomes challenging when heterozygosity exceeds 0.5% – 1% (Marcais and Kingsford, 2011), but *G. prostrata’s* heterozygosity is 0.159%, indicating simplicity. With 34.6% repetitive sequences, below the 50% threshold for complexity, *G. prostrata* has a low complexity genome (Sun et al., 2023). In addition, GC content distribution is important for assessing the quality of genome sequencing and the complexity of genome assembly (Singh et al., 2016). By analyzing the GC-depth distribution plot, we can determine if there’s a notable GC bias in the sequencing data (Benjamini and Speed, 2011) and identify potential bacterial contamination (Parras-Molto et al., 2018). Not only that, GC content, crucial for successful assembly, is ideal between 25%–65% (Aird et al., 2011). *G. prostrata*’s GC content of 38.85% is within this range, comparable to *Rosa roxburghii* (38.5%) and *Helianthus annuus* (38.9%) *(* (Bi et al., 2019; Shan et al., 2020). It could be seen that the GC content of *G. prostrata* is at a moderate level, which has been demonstrated to be suitable for assembly. At the same time, the scaffold N50 lengths of it exceeded 30 kb (Nagarajan and Pop, 2013), and the genome completeness estimated by BUSCO using the embryophyta_odb10 dataset is 90%, indicating that the *G. prostrata* genome is a high-quality assembly result.

Significantly, genome size, GC content, and repetitive sequences influence genome complexity, assembly, and species adaptability. Genome size is influenced by a variety of biological factors, including the cell cycle, cell dimensions, and stress tolerance (Wan et al., 2021). In addition, in some cases, there is a correlation between genome size and plant body size (Leitch and Bennett, 2004). In some taxa, genome size correlates with body size, aiding in faster cell growth in limited resources (Greilhuber and Leitch, 2012). However, in other plants, this correlation does not hold. *G. prostrata*’s genome (410 Mb) is smaller than related Rhododendron species (~ 560 - 650 Mb) (Zhang et al., 2017; Soza et al., 2019; Yuan et al., 2019) and moderate among high-altitude plants (Yang et al., 2013), potentially reducing evolutionary costs (Cobo-Simon and Tamames, 2017). Besides, reduced leaf area and height help minimize heat loss and provide cold protection. Meanwhile, genome size is positively correlated with GC content and higher GC content generally confers greater thermostability and flexibility to DNA (Vinogradov, 2003). *G. prostrata*’s higher GC content compared to *A. thaliana* (Wright et al., 2002) improves DNA stability and reduces DNA curvature, aiding in high-altitude adaptation (Vinogradov, 2003). In *G. prostrata*, moderate GC content [compared with *R. indicum* (39%) and *R. micranthum* (40.4%) (Yuan et al., 2019; Zhou et al., 2020)] likely balances sufficient chromatin openness with the necessary flexibility for effective gene expression, thereby ensuring the stability and efficiency of physiological and metabolic processes. Moreover, repetitive sequences play a significant role in the regulation of structural genes and the control of recombination processes (Shi et al., 2016a). They enhance genomic diversity and adaptability by triggering gene recombination, mutation, and new gene formation (Shi et al., 2018). From another perspective, our study revealed that significant variations in the frequencies of various types of SSR repeats, with higher frequencies of di-, mono-, and trinucleotide repeats in *G. prostrata*. This result parallels findings in most plants (Shan et al., 2020; Reinar et al., 2021). The high proportion of dinucleotide SSRs in plant genomes, particularly AG/CT motifs, suggests their crucial role in genome stability, evolution, and as markers for genetic diversity due to their high mutation rates and polymorphism (Niyitanga et al., 2022). In summary, the abundance of SSRs in *G. prostrata* likely enhances its genetic diversity, supporting a wide array of physiological adaptations. This diversity may facilitate rapid evolution of traits, such as improved photosynthetic efficiency to withstand high UV exposure or enhanced antifreeze proteins for cold resistance (Selkoe and Toonen, 2006; Yocca et al., 2023). which is a key factor in its successful colonization and reproduction in diverse and extreme alpine environments. Overall, the unique genomic characteristics of *G. prostrata*, including its optimized genome size, moderate GC content, and abundant SSRs, enable it to thrive under the severe and fluctuating conditions of high-altitude environments.

Furthermore, among the TFs, TRs, and PKs we identified, the top ten genes in each category are involved in regulating environmental adaptation, responding to biotic and abiotic stresses (Zhu, 2016), and mediating light signal transduction (Chen et al., 2004). This regulation can promote the loosening of chromatin in specific gene regions, making these genes more easily transcribed (Li et al., 2007). By regulating gene expression, plants can respond quickly to environmental changes, thereby helping *G. prostrata* better adapt to high-altitude habitats. Then, our study identified 26,497 protein-coding genes focused on environmental stress response, notably in areas like plant-pathogen interaction and hormone signal transduction, using GFAP across multiple databases. These genes, active in transcription regulation and defense, suggest robust immune mechanisms evolved against microbial threats. These genes, active in transcription regulation and defense, suggest robust immune mechanisms evolved against microbial threats. It is noteworthy that *G. prostrata* exhibits a rich composition of methyl salicylate, which is also involved in stress resistance (Liu et al., 2011). Future research could explore genes regulating methyl salicylate biosynthesis in stress responses. Moreover, the MAPK signaling pathway is a critical component of the plant response to external stressors, including pathogens and abiotic stress (Cristina et al., 2010). It plays a crucial role in some plant’s response to the fluctuating environmental conditions typically found at high altitudes. The MAPK pathway is involved in regulating cellular responses to temperature fluctuations, UV radiation, and other abiotic stresses, thereby enhancing the plants’ survival and growth in these challenging environments (Liu et al., 2022; Kumar et al., 2023; Joshi et al., 2024). While the transcription regulation genes, especially those that are DNA-templated, play a central role in orchestrating a diverse array of stress responses (Jiang et al., 2017).

Lu et al.’s research indicates that the origin of *Gaultheria* Kalm *e*x L. (Ericaceae) dates back to approximately 20–25 million years ago (Lu et al., 2019), which coincides with our estimated origin for *G. prostrata* at 21.48 million years. This period is belong to the early Miocene epoch, when the Earth experienced significant climatic changes, including initial warming followed by cooling; the expansion of grasslands driven by increased aridity; major tectonic activities, such as the uplift of the Himalayas; and considerable biological diversification among plants and mammals (Boyer, 2023; Britannica, T.E.o.E, 2023; Paleontology, UCMP, 2023). These changes may have created new ecological opportunities or adaptive pressures for the *Gaultheria* plants.

The identified gene of expansion, contraction, and positive selection in *G. prostrata* highlight its adaptation to high-altitude environments. Gene contraction, particularly in secondary metabolite synthesis and light response pathways, suggests a streamlined metabolism for survival in resource-limited conditions, while the expansion of genes related to the MAPK signaling pathway and transmembrane transport enhances its stress response capabilities. In contrast, *V. corymbosum* shows substantial gene expansion, indicating different evolutionary pressures. Additionally, positive selection of genes involved in immune and osmotic stress responses further supports *G. prostrata*’s adaptation to extreme environments. Understanding how these genes and pathways help high-altitude plants adapt to harsh environments can provide potential applications for agriculture and ecological restoration (Schranz et al., 2012). For example, these genes can be targeted by gene editing or breeding programs to develop crops that are more resistant and environmentally adaptable (Chen et al., 2019a). This is of great significance for responding to climate change and improving crop tolerance and stability, especially for applications in agricultural marginal areas or extreme environments (Bailey-Serres et al., 2019). Although we have identified significantly enriched pathways, further functional validation and experimental studies are needed to reveal the specific roles of these genes in high altitude acclimatization. Future studies could focus on the experimental validation of the function of the genes, environmental simulation experiments, and how these genes are dynamically regulated under different environmental stresses (Burtscher et al., 2022). In addition, the synergistic effects of gene networks and multiple pathways can be explored to fully understand the complex regulatory mechanisms of *G. prostrata* in response to environmental changes.

In conclusion, future studies should integrate second- and third-generation sequencing for the complete genome sequencing of *G. prostrata*. Employing Hi-C technology will aid in understanding its chromosome organization and regulatory mechanisms, essential for utilizing its germplasm resources. T2T (Telomere-to-Telomere) technology can reveal genetic details missed by traditional sequencing, especially in complex region. Third-generation genome data will enhance comparisons and insights into *Gaultheria*’s genetic and evolutionary traits. In the future, integrating phylogenomic and environmental data will help reconstruct the evolutionary history of *Gaultheria* and reveal key biogeographic and adaptive patterns. Simultaneously, future research could also investigate those metabolites that are related to the high-altitude adaptability of *G. prostrata*, aim to identify characteristic metabolites, and determine whether there are some repetitive sequences or other genomic structures that respond adaptively to environmental stress.

## Data Availability

The datasets presented in this study can be found in online repositories. The names of the repository/repositories and accession number(s) can be found below: https://www.ncbi.nlm.nih.gov/, PRJNA1042922.
