# Supplementary figures and images for: First De Novo genome assembly and characterization of Gaultheria prostrata
Source: Front Plant Sci. 2024 Oct 29;15:1456102. doi: 10.3389/fpls.2024.1456102 (PMC11554542; doi:10.3389/fpls.2024.1456102)

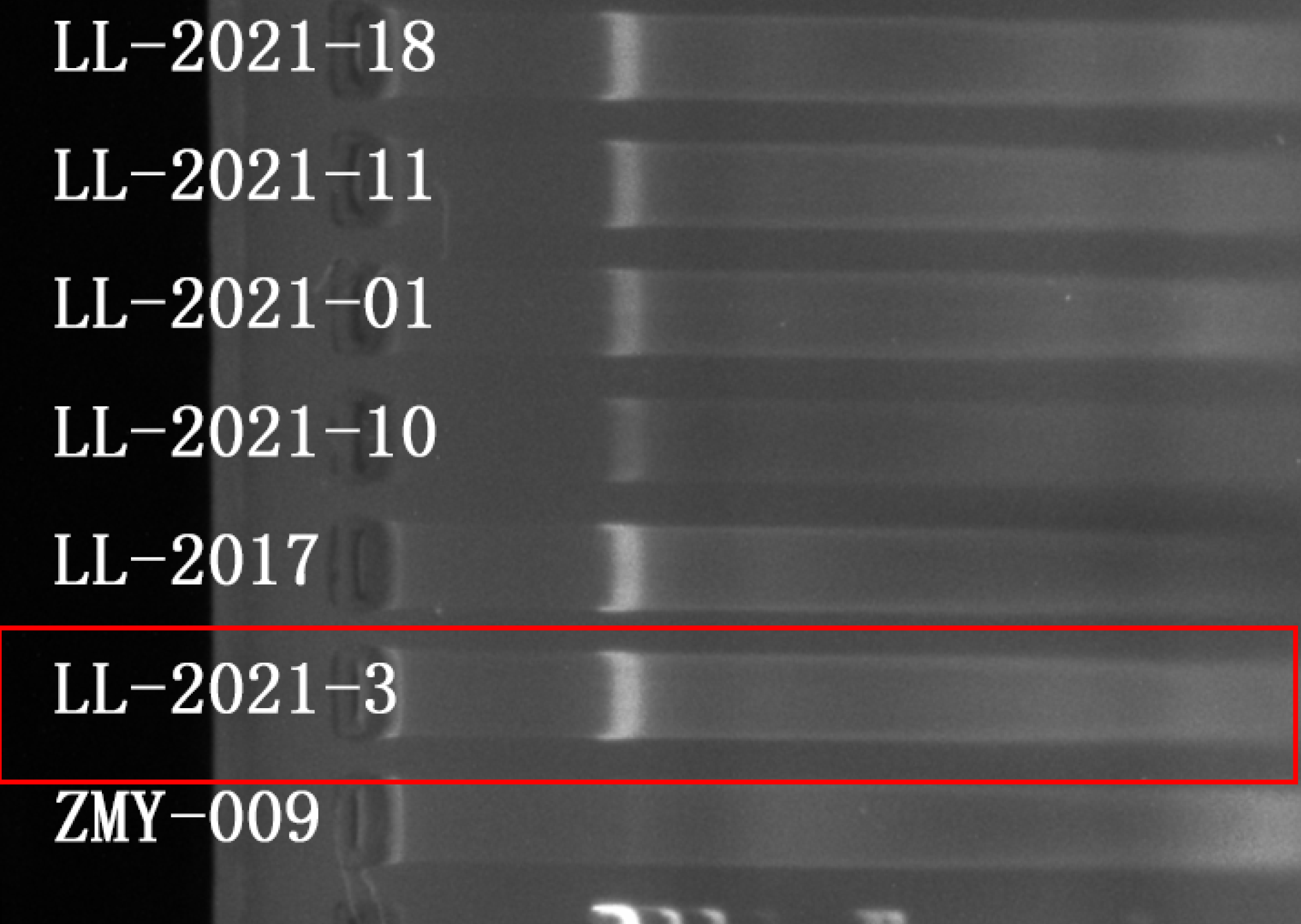

Supplement: Supplementary file 1 [file Image1.tif]

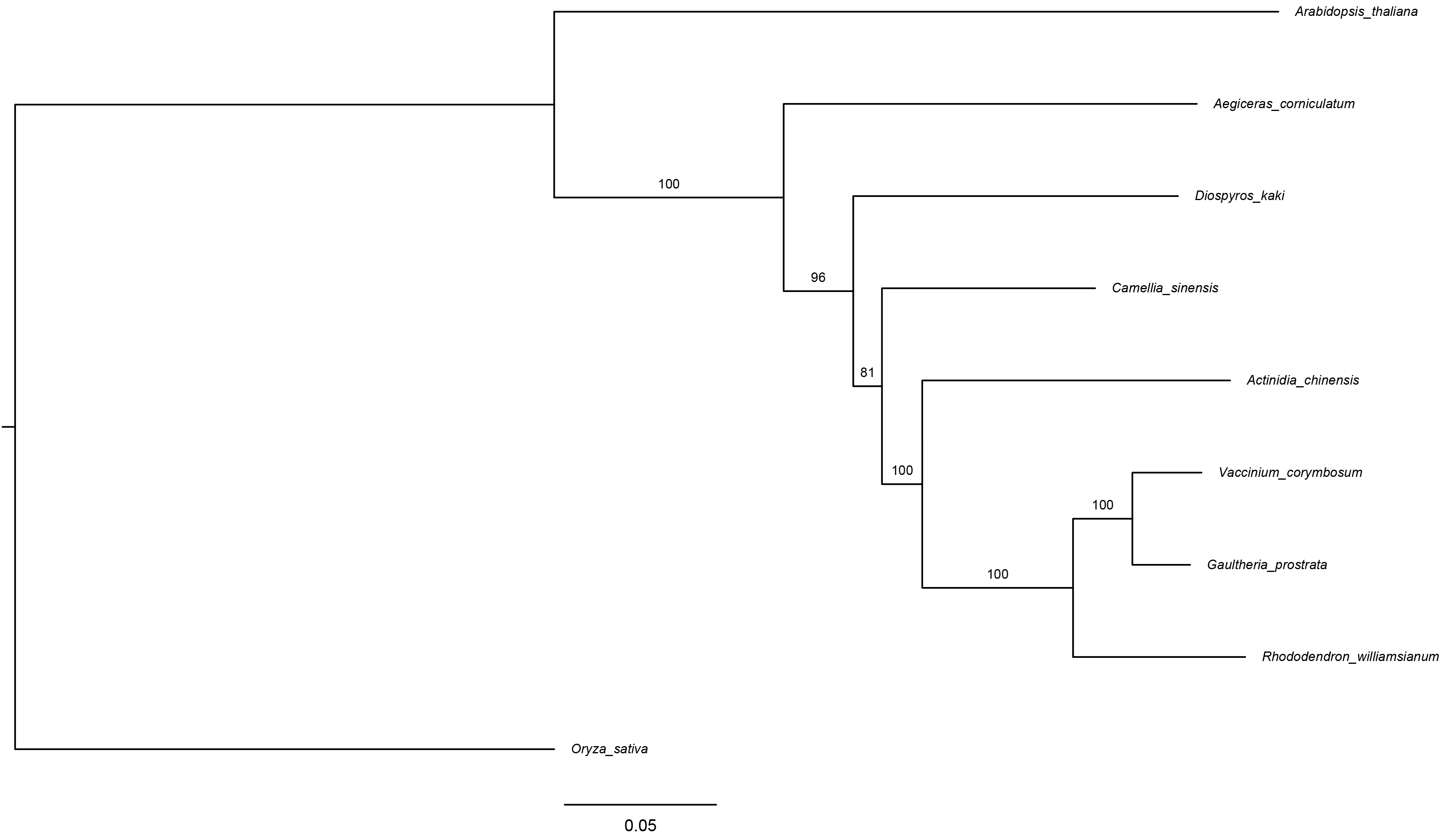

Supplement: Supplementary file 2 [file Image2.tif]

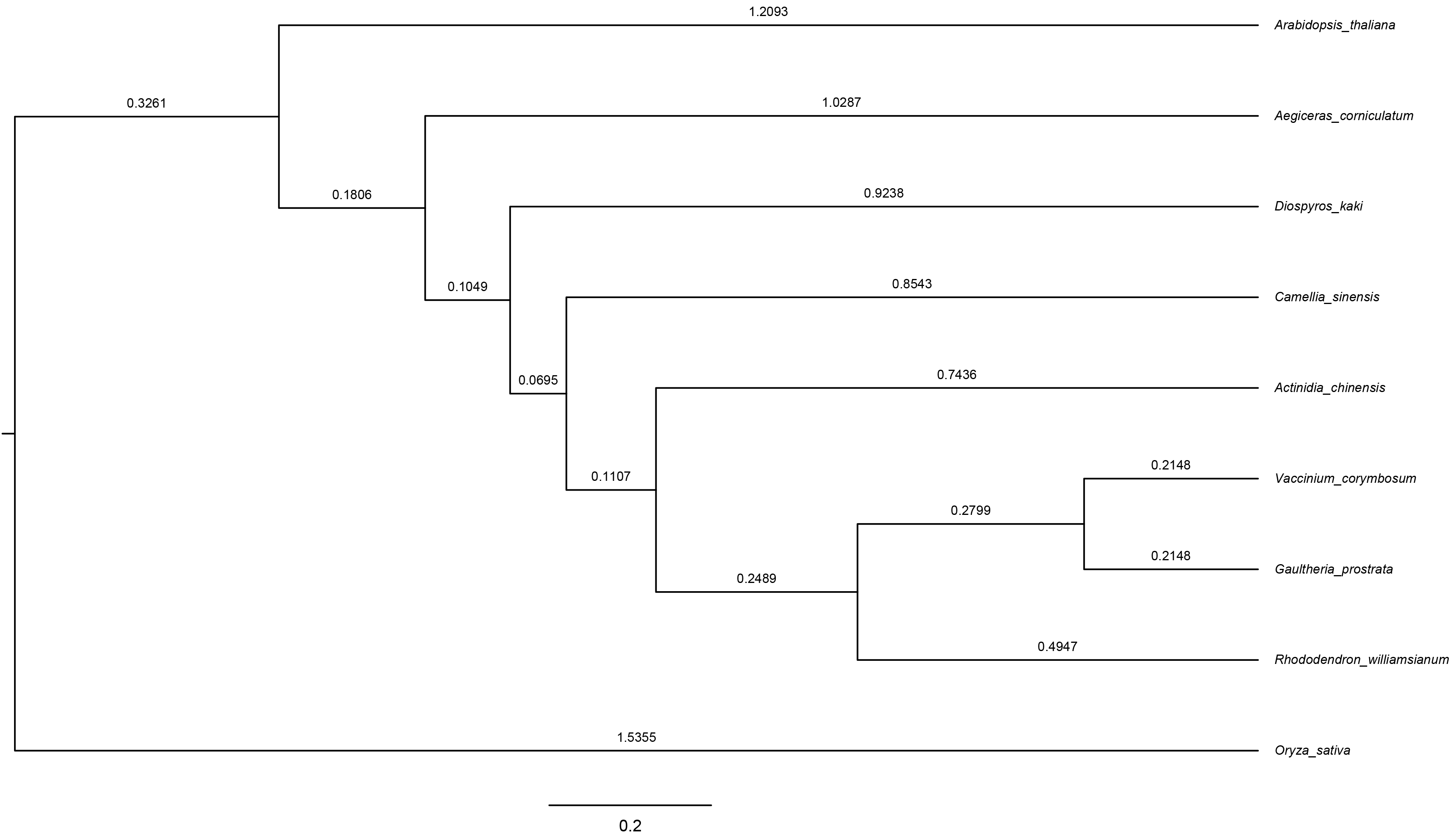

Supplement: Supplementary file 3 [file Image3.tif]
